# Comparisons of pharmacokinetics of glimepiride in combination with Ojeok-san versus glimepiride alone: an open-label, one-sequence, two-treatment controlled clinical study

**DOI:** 10.1038/s41598-025-09317-z

**Published:** 2025-07-16

**Authors:** Jongyoon Kim, Minji Kwon, Sooyoung Lee, Jeein Noh, Wang-Seob Shim, Eunseo Song, Kyung-Tae Lee, Ji-Young Park, Sung-Vin Yim, Bo-Hyung Kim

**Affiliations:** 1 Department of Medicine, Graduate School, Kyung Hee University, Seoul, 02447 Republic of Korea; 2 Department of Clinical Pharmacology and Therapeutics, Kyung Hee University Hospital, Seoul, 02447 Republic of Korea; 3 East-West Medical Research Institute, Kyung Hee University, Seoul, 02447, Republic of Korea; 4 Department of Regulatory Science, Graduate School, Kyung Hee University, Seoul, 02447 Republic of Korea; 5 Kyung Hee Drug Analysis Center, College of Pharmacy, Kyung Hee University, Seoul, 02447 Republic of Korea; 6 Department of Biomedical and Pharmaceutical Sciences, Graduate School, Kyung Hee University, Seoul, 02447 Republic of Korea; 7 Department of Pharmaceutical Biochemistry, College of Pharmacy, Kyung Hee University, Seoul, 02447 Republic of Korea; 8 Department of Clinical Pharmacology and Toxicology, Anam Hospital, Korea University College of Medicine, Seoul, 02841 Republic of Korea; 9 Department of Clinical Pharmacology and Therapeutics, College of Medicine, Kyung Hee University, Seoul, 02447 Republic of Korea; 10 Department of Biomedical Science and Technology, Graduate School, Kyung Hee University, Seoul, 02447 Republic of Korea

**Keywords:** Glimepiride, Ojeok-san, Herb-Drug interaction, Pharmacokinetics, Health care, Medical research

## Abstract

Glimepiride remains a cost-effective antidiabetic treatment despite its potential risks. However, its interaction with traditional medicines like Ojeok-san (OJS), a commonly used herbal medication, warrants investigation. This open-label, fixed-sequence, two-period, two-treatment crossover study involved 17 healthy male volunteers. Subjects received glimepiride 4 mg once daily for 2 days in period 1, followed by OJS 4.35 g three times daily for 8 days, with concurrent glimepiride administration on the final two days in period 2. Co-administration of OJS with glimepiride resulted in pharmacokinetic changes. The mean area under the plasma concentration-time curve (AUC) from dosing to 24 h post-dosing (AUC_0–24 h_) of glimepiride decreased from 1283.53 ng∙h/mL to 1125.27 ng∙h/mL, and the mean maximum concentration (C_max_) reduced from 250.76 ng/mL to 209.38 ng/mL when compared to glimepiride alone. OJS co-administration also prolonged the median time to reach maximum concentration (T_max_) and half-life (t_1/2_). The study demonstrated pharmacokinetic interactions between glimepiride and OJS, showing reduced systemic exposure and altered elimination patterns of glimepiride during co-administration.

**Supplementary Information:**

The online version contains supplementary material available at 10.1038/s41598-025-09317-z.

## Introduction

Type 2 diabetes mellitus (T2DM) has emerged as a significant global public health concern with an increasing prevalence in recent decades. As of 2021, the International Diabetes Federation has reported a global diabetes prevalence of 10.5%, impacting 537 million adults aged 20–79 years^1^. T2DM comprises around 90% of all diabetes cases and is increasing globally^2^. Regional disparities exist, with East and South Asia facing the largest rise, while Northwestern Europe exhibits the smallest prevalence increases^2^.

Glimepiride is a second-generation sulfonylurea used to treat T2DM. It stimulates insulin release from the pancreatic beta cells, leading to a decrease in blood glucose levels^3^. Sulfonylurea drugs, such as glimepiride, has been shown to be effective in reducing Hemoglobin A1C (HbA1c) levels, and fasting plasma glucose levels in patients with T2DM^4^. It is anticipated to be comparably efficacious to other sulfonylureas in mitigating the risk of microvascular complications, including retinopathy^4^. Despite a global decline in the use of sulfonylureas owing to its potential drawbacks, such as an elevated risk of hypoglycemia and controverted cardiovascular safety, compared with other available antidiabetic medications^5^, its continued use as second-line treatment may be attributed to its cost^6^. The utility of glimepiride remains significant, especially in India, where glimepiride is commonly prescribed as fixed-dose combination formulation with metformin and voglibose for T2DM^7^. Also, the World Health Organization (WHO) guideline in low-resource settings recommend that a sulfonylurea is given with T2DM patients who do not achieve glycemic control with metformin alone or who have contraindications to metformin^8^.

Following oral administration, glimepiride, a sulfonylurea drug, is rapidly absorbed, reaching peak plasma concentrations within 2–3 h. Absorption of certain sulfonylurea drugs from the intestinal lumen occurs via OATP2B1 transporters present in intestinal cells^9^. Glimepiride undergoes extensive hepatic metabolism primarily by cytochrome P450 enzyme CYP2C9, with OATP1B1 facilitating hepatic uptake^3,10^. Metabolites are excreted mainly via the kidneys, with approximately 60% recovered in the urine and the remainder portion eliminated via feces^11^. Several factors can affect the PKs of glimepiride, including age, sex, race, and the concomitant use of other drugs that induce or inhibit CYP2C9. For example, co-administration of rifampin, a potent CYP2C9 inducer, can significantly reduce plasma concentrations of glimepiride, whereas co-administration of fluconazole, a potent CYP2C9 inhibitor, can increase plasma concentrations of glimepiride^12,13^.

According to the WHO, a significant portion of the global population relies on indigenous or traditional forms of medicine for routine healthcare needs^14^. In developed countries such as Europe and the United States, herbal medicines constitute approximately 25% of all medications. A randomized survey conducted in Australia in 2007 revealed that more than two-thirds of participants (68.9%) had used complementary and alternative medicine in the previous 12 months, showing a higher prevalence than previously estimated in regional studies^15^. Similarly, preliminary studies in New Zealand have reported substantial usage of traditional, complementary, and alternative medicine^16^. In recent years, traditional medicine, including East Asian herbal remedies, has gained widespread acceptance in Western medical communities, with the general public increasingly integrating these approaches to meet diverse health needs.

Ojeok-san (OJS), also known as Wuji-san and Goshaku-san, is one of the most widely used herbal medicines in Korea, Japan, and China^17,18^. This traditional herbal medicine has been used for centuries to treat various disease as an analgesic^19^. Recent clinical trials have investigated its efficacy in treating conditions such as cold hypersensitivity in hands and feet among women and chronic cough associated with gastroesophageal reflux^18,20^. Due to its diverse pharmacological effects, OJS is known to be the most frequently prescribed herbal medicine among those covered by the national insurance system in Korea. According to insurance claims data from 2019 to 2023, OJS accounted for approximately 3.5 to 4.2 million claims annually, the highest among reimbursed herbal formulas^21^. Therefore, in patients with chronic diseases such as diabetes, there is a potential for short-term co-administration of OJS with conventional medications, including hypoglycemic agents. This highlights the need to evaluate possible interactions and the effects of such co-administration.

The medicine is made up of a combination of 17 different herbs, including *Angelicae Gigantis Radix*, *Paeoniae radix*, *Cnidii rhizome*, *Zingiberis Rhizoma*, and *Cinnamomi Cortex*, etc^19^. Analytical markers identified in OJS include gallic acid, paeoniflorin, ferulic acid, hesperidin, naringin^19^. Studies have demonstrated its therapeutic effects, including anti-inflammatory, analgesic, and immunomodulatory properties.

Several pharmacokinetic (PK) studies have identified various active components of OJS in the bloodstream after oral administration, including gallic acid, *Paeoniae Radix* extract, and ferulate^22–24^. Among the identified analytical markers, gallic acid, chlorogenic acid, ferulic acid, hesperidin, naringin, and cinnamic acid are classified as phenolic compounds (PCs)^25^. Gallic acid, naringin and ferulic acid among these PCs have been reported to exhibit herb or nutrient-drug interactions^25^. Undesirable effects may arise from herb-drug combinations when the herbal product alters the activity of enzymes involved in drug metabolism and/or transporters responsible for drug distribution^26^. The concurrent administration of OJS is capable of altering the PK characteristics of medications metabolized by these enzymes. Consequently, the co-administration of oral hypoglycemic agents with OJS may necessitate dosage modifications to mitigate PK interactions. Moreover, considering the pharmacodynamic (PD) aspect, a retrospective analysis in T2DM patients suggested that OJS may have a hypoglycemic effect^27^. In hospitalized patients, fasting blood sugar (FBS) and 2-hour postprandial glucose (PP2) levels improved after the coadministration of OJS and anti-diabetic drugs compared to baseline values prior to OJS administration. However, this effect on PP2 was not significant in patients with HbA1c below 6.5%. Although these results may reflect dietary control or regular antidiabetic medication use, the potential glucose-lowering effect of OJS cannot be excluded. Thus, further evaluation is needed to assess whether co-administration of OJS with antidiabetic drugs affects glycemic control through changes in drug levels or other pharmacological mechanisms.

Therefore, consideration of the potential drug interactions between glimepiride and OJS is important to ensure patient safety and optimize treatment outcomes. This interaction should be quantitatively evaluated based on the PK results between glimepiride and OJS because the effective dosage and regimens of glimepiride should be optimized using these results. Therefore, an herb-drug interaction study was planned to compare the outcomes of glimepiride alone and the co-administration of glimepiride and OJS in healthy volunteers.

## Results

### Demographics

Of the 22 volunteers screened, 17 healthy male volunteers met the eligibility criteria and were enrolled in the study. One volunteer withdrew consent immediately after hospitalization in period 1, prior to receiving glimepiride. Among the 16 participants who completed the study schedule at period 1, three withdrew consent prior to drug administration in the period 2. Therefore, 13 participants successfully completed the study.

The mean ± standard deviation (min, max) of age, weight, height, and body mass index (*n* = 17) were 30.59 ± 7.58 (21, 45) years, 72.47 ± 9.74 (54.2, 88.9) kg, 173.42 ± 5.6 (166.1, 182.6) cm, 24.08 ± 2.83 (18.01, 27.29) kg/m^2^, respectively. The safety profile assessment included 16 subjects who received at least one dose of the investigational drug and one subject who was excluded because of withdrawal of consent prior to dosing. In the case of PK and PD analyses, all data measured until the last time point were used.

### Pharmacokinetics

**Fig. 1:**
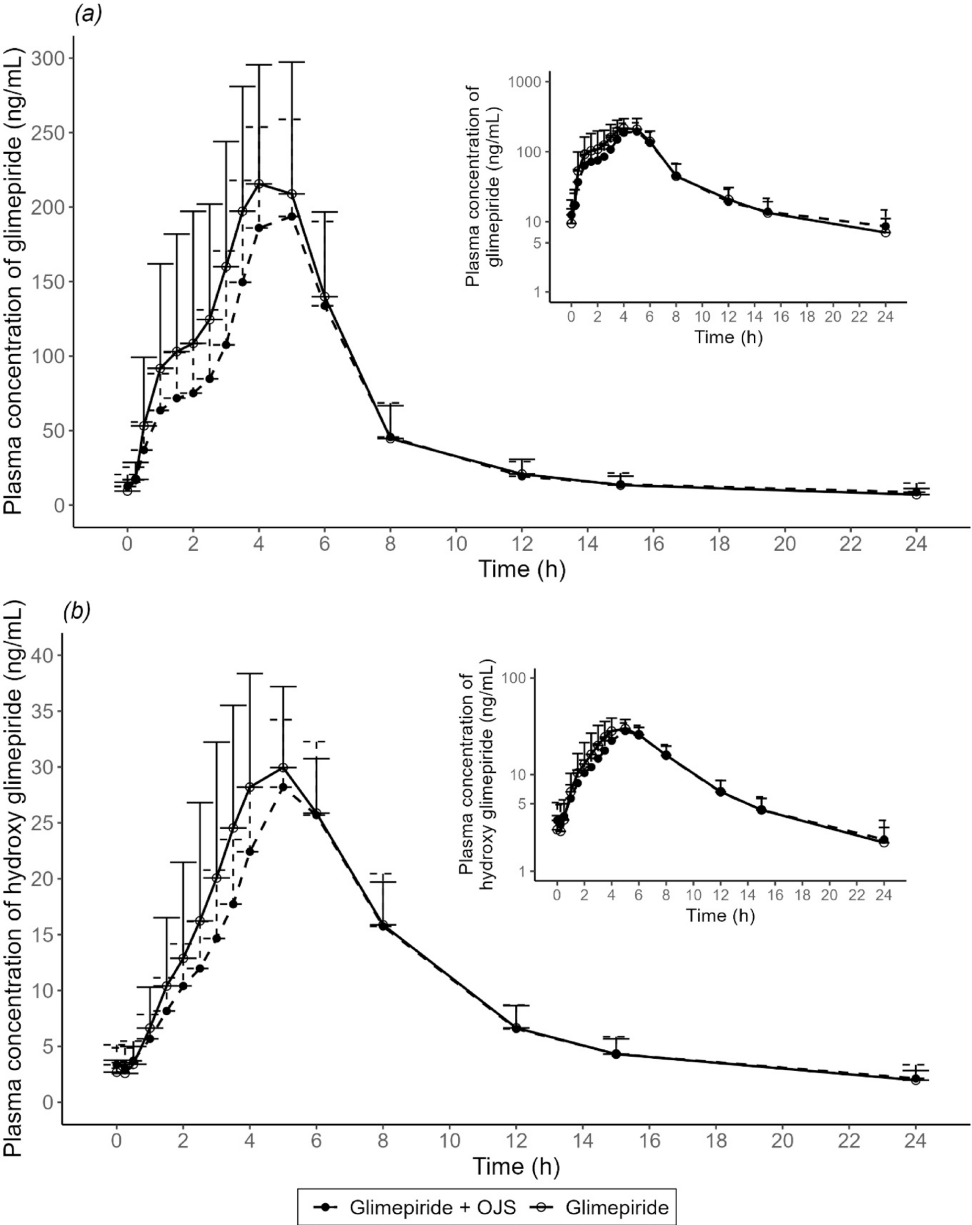
Mean plasma concentration-time curves from 0 to 24 h for (**a**) glimepiride and (**b**) hydroxy glimepiride following monotherapy and combination therapy with OJS. Error bars indicate the standard deviations. OJS, Ojeok-san.

The mean plasma concentration profiles of glimepiride and M1 show that OJS decreased the absorption of glimepiride and M1 (Fig. 1, Supplementary Fig. S1). The presence of OJS prolonged the median T_max_ of glimepiride from 4.3 to 5.0 h (Table 1). The mean AUC_0–24 h_ of glimepiride decreased from 1283.53 to 1125.27 ng∙h/mL, when the co-administration of both drugs compared to glimepiride alone. Additionally, mean C_max_ of glimepiride decreased from 250.76 to 209.38 ng/mL. However, mean t_1/2_ of glimepiride increased from 8.53 to 10.16 h (Table 1). Supplementary material includes graphs following administration of either glimepiride alone or in conjunction with OJS over a 0–48 h period (Supplementary Fig. S2).

**Table 1:** Comparative pharmacokinetic parameters of glimepiride following concurrent administration with glimepiride and Ojeok-san versus administration with glimepiride.

		Total (*n* = 16)	
	Glimepiride + OJS (*N* = 13)	Glimepiride (*N* = 16)	Geometric mean ratio^a^ (%, 90% CI)
Glimepiride			
T_max_ (h)	5.0 (3.5–5.05)	4.5 (2.0–5.0)	
C_max_ (ng/mL)	209.38 ± 71.41	250.76 ± 86.99	83.14 (69.96–98.81)
AUC_0–24 h_ (ng∙h/mL)	1125.27 ± 406.43	1283.53 ± 482.89	86.43 (77.56–96.31)
AUC_0–48 h_ (ng∙h/mL)	1160.26 ± 467.61	1304.83 ± 518.95	86.98 (77.49–97.63)
AUC_inf_ (ng∙h/mL)	1279.12 ± 551.28	1379.93 ± 506.81	89.15 (81.72–97.26)
t_1/2,0–24 h_ (h)	10.16 ± 4.84	8.53 ± 4.69	
t_1/2,0–48 h_ (h)	9.33 ± 3.02	8.73 ± 4.78	
CL/F (L/h)	3.68 ± 1.54	3.28 ± 1.16	
M1			
T_max_ (h)	5.0 (5.0–6.0)	5.0 (3.0–6.0)	
C_max_ (ng/mL)	28.75 ± 6.40	32.61 ± 8.08	85.33 (77.77–93.62)
AUC_0–24 h_ (ng∙h/mL)	221.98 ± 49.56	237.85 ± 61.69	91.77 (85.53–98.46)
AUC_0–48 h_ (ng∙h/mL)	229.57 ± 58.46	243.05 ± 70.10	92.48 (85.29–100.26)
AUC_inf_ (ng∙h/mL)	250.87 ± 68.00	258.17 ± 67.99	95.05 (89.10–101.40)
t_1/2,0–24 h_ (h)	8.04 ± 2.95	6.73 ± 1.35	
t_1/2,0–48 h_ (h)	8.02 ± 2.80	7.08 ± 1.97	
CL/F (L/h)	17.13 ± 4.89	16.40 ± 4.00	

Data are summarized as the arithmetic mean ± standard deviation, except for T_max_, which is presented as the median (min–max). OJS, Ojeok-san; CI, confidence interval; T_max_, time to maximum plasma concentration; C_max_, maximum plasma concentration; AUC, area under the concentration-time curve; t_1/2_, terminal half-life; CL/F, apparent clearance; M1, hydroxy glimepiride.

^a^Geometric mean ratio of glimepiride and OJS co-administration to glimepiride administration alone.

**Fig. 2:**
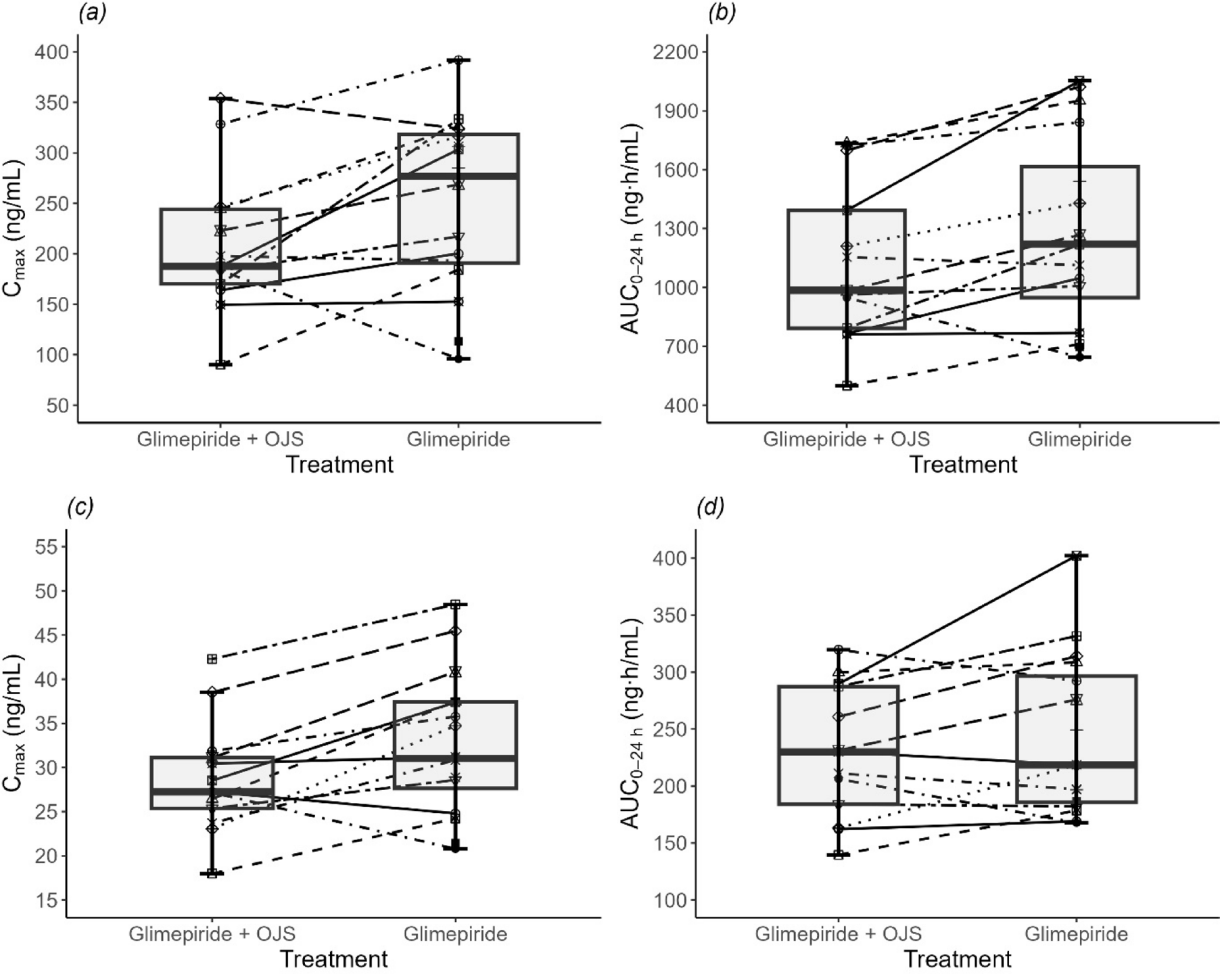
Individual (**a**) glimepiride C_max_, (**b**) glimepiride AUC_0–24 h_, (**c**) hydroxy glimepiride C_max_, (**d**) hydroxy glimepiride AUC_0–24 h_ when glimepiride was administered alone or in combination with Ojeok-san. Box plots represent the lower quartile, median, and upper quartile. C_max_, maximum concentration; AUC_0–24 h_, area under the concentration time curve during the dosing interval; OJS, Ojeok-san.

The co-administration generally resulted in a decrease in the C_max_ and AUC_0–24 h_ for both glimepiride and its metabolite M1, relative to the administration of glimepiride in isolation (Fig. 2). The GMR (%, 90% CI) of C_max_ and AUC_0–24 h_ for glimepiride was 83.14 (69.96, 98.81) and 86.43 (77.56, 96.31), which were outside the range of 0.8 to 1.25 (Table 1). Meanwhile, the GMR (%, 90% CI) of C_max_ and AUC_0–24 h_ for M1 was 85.33 (77.77, 93.62) and 91.77 (85.53, 98.46), respectively, indicating that the reduction in the systemic exposure of M1 was less than that of glimepiride. The metabolic ratios were not significantly different between the co-administration and glimepiride-alone groups (*p* = 0.1209).

To investigate the influence of genetic polymorphisms on individual subjects’ CYP2C9-mediated metabolism, plasma drug concentrations, and drug-drug interactions, an analysis of CYP2C9 genotypes in 13 subjects revealed a uniform presence of the wild-type allele, CYP2C9*1/*1 across all participants.

### Safety and tolerability

During period 1, 6 out of 16 subjects (37.5%) reported adverse events (AEs), while during period 2, 2 out of 13 subjects (15.4%) reported AEs. All AEs were related to glimepiride administration and have been previously reported in other studies. AEs documented in relation to glimepiride encompassed a solitary instance of dizziness and five occurrences of hyperhidrosis during the initial study phase (Period 1), whereas two instances of hyperhidrosis were noted during the subsequent study phase (Period 2). None of the participants withdrew from the clinical study because of AE. Safety profiles and tolerability assessments, including vital signs, physical examinations, ECGs, and clinical laboratory tests, showed no significant changes from baseline.

## Discussion

In this study, the concomitant use of OJS reduced systemic exposure to glimepiride, as indicated by the observed decrease in C_max_ and AUC. However, the mean t_1/2_ of glimepiride was prolonged. These findings suggested that the simultaneous use of OJS influenced both the absorption and elimination phases of glimepiride, consequently affecting its PKs. During the absorption phase, the mean C_max_ of glimepiride and its metabolite M1 were reduced by approximately 16.86% and 14.67%, respectively, compared to glimepiride monotherapy. Conversely, in the elimination phase, the mean t_1/2, 0–24 h_ of glimepiride treatment was prolonged by approximately 1.63 h (1.31 h for M1) in the presence of OJS. These findings collectively indicate that while both the elimination rate and absorption of glimepiride were diminished in the presence of OJS, the magnitude of the PK interactions between OJS and glimepiride was notably greater during the absorption phase than during the elimination phase.

Glimepiride was administered at a dose of 4 mg once daily for two consecutive days, rather than as a single dose, while OJS was administered at 4.35 g per pack, three times daily for 8 days in the present study. The two-day dosing regimen for glimepiride was adopted based on the design of a previous PK interaction study involving glimepiride and evogliptin^28^. The mean elimination half-life of glimepiride has been reported to range from 7.37 to 8.55 hours^29^ with a value of 8.5 h observed in the current study. Although steady-state conditions were not projected to be fully achieved with the two-day dosing period, the mean AUC was anticipated to exceed 90% of the steady-state exposure. Consequently, the two-day dosing was deemed reasonable for approximating steady-state concentrations for the purposes of this study. The eight-day administration period for OJS was selected based on its typical short-term clinical use, generally lasting from several days to up to two weeks when used as an analgesic and other therapeutic purpose. In addition, this duration was considered suitable for evaluating potential drug-herb interactions, including the induction of drug-metabolizing enzymes, consistent with the design of previous study^30^. Regarding the PK sampling schedule, the plasma concentration at 48 h were measured due to the possibility of glimepiride-OJS interaction. However, in all subjects except three, concentrations of glimepiride and M1 measured at 48 h were below the LLOQ. Specifically, those at 48 h were above the LLOQ for two subjects when administered glimepiride alone and two subjects when co-administered glimepiride with OJS. Therefore, it was judged that the differences in AUC_0–48 h_ and t_1/2,0–48 h_ between glimepiride alone and glimepiride with OJS were not significant.

The glimepiride prescribing information indicates that mean C_max_ and AUC when glimepiride is administered with a meal decreased by 8% and 9%, respectively^31^. Similarly, when glimepiride was co-administered with aspirin, mean C_max_ and AUC were reduced by 4% and 34%, respectively^31^. Additionally, colesevelam, a bile acid sequestrant, impaired the absorption of glimepiride, i.e. reductions of glimepiride C_max_ and AUC (8% and 18%, respectively)^32^. In another study, co-administration of celecoxib with OJS decreased celecoxib C_max_ and AUC by 27.5% and 11.5%, respectively^30^. These findings suggest that OJS may affect the absorption of glimepiride in a similar way to food or bile acid sequestrants or alter environment in the absorption site, i.e. change of pH or delaying gastric emptying time and so on.

Among the analytical markers of OJS, polyphenolic compounds such as naringin, and gallic acids are known to primarily influence the inhibitory activities of CYP2C9 and CYP3A4^24,33^. This behavior of the CYP2C9 and CYP3A4 enzymes may explain PK changes due to the drug-herb interaction observed with the co-administration of celecoxib in the previous study or glimepiride in the current study^30^. Celecoxib is a CYP2C9 and CYP3A4 substrate, and glimepiride is a CYP2C9 substrate. The prolongation in t_1/2_ of celecoxib and glimepiride following co-administration of OJS can be interpreted as being primarily mediated by the CYP2C9 enzyme. In addition to the CYP inhibitory mechanisms of PCs, the inhibitory effects of OJS on human microsomal CYP450 enzymes should be evaluated. In this regard, a previous study has shown OJS to be a weak competitive inhibitor of CYP450 enzyme activity, including CYP2C9 and CYP3A4, with IC50 values of 868.74 and 583.60 µg/mL, respectively^34^. Therefore, the prolongation of glimepiride t_1/2_ observed with OJS co-administration in the current study could be explained by these in vitro findings.

This study aimed to elucidate the influence of CYP2C9 polymorphisms, which play a pivotal role in the metabolic processing of glimepiride. Genotypic analysis conducted in this study showed that all 16 participants were homozygous for the wild-type allele of CYP2C9 (CYP2C9*1/*1), thereby demonstrating the conventional enzymatic functionality of CYP2C9. Previous studies have reported that the CYP2C9*2 is not found within the Korean demographic, and the frequency of CYP2C9*3 allele were 0.044% and 0.04%^35,36^. Exclusively wild-type genotypes in the current study were identified within this participant pool, a phenomenon attributable to the constrained sample dimension.

Glimepiride absorption occurs through hepatic uptake by OATP1B1 transporter^10^. Since hesperidin and naringin act as inhibitors of OATP1B1^37^, drug-herb interactions involving this transporter are likely. However, both substrates, as well as other herbal components of OJS, may induce OATP1B1 expression, similar to the findings observed with berberine^38^. This study results demonstrated a marked decrease in blood concentration during the absorption phase, which may be attributed to enhanced hepatic uptake of glimepiride via OATP1B1 following prior repeated administration of OJS.

Due to technical error, OGTTs were administered with an intake of 50 g of glucose on the first and second days, followed by a dose of 75 g on the ninth and tenth days. The analysis of glycemic responses to a reduced glucose load of 25 g in evaluating the glycemic control provided by glimepiride monotherapy, as opposed to the concomitant administration of glimepiride and OJS, was constrained.

In a previous study, healthy Korean participants assessed post-OGTT glucose responses following a 75 g glucose load, reporting the baseline mean AUG_0–3 h_ and G_max_ values of 392 ± 66.7 mg∙h/dL and 174.8 ± 34.2 mg/dL, respectively^39^. In another study involving young, healthy Korean individuals, participants first consumed 75 g of glucose, then received 4 mg of glimepiride, followed by another 75 g glucose load^28^. After administering 75 g of glucose, baseline mean AUG_0–3 h_ and G_max_ were reported as 384 ± 65.9 mg∙h/dL and 175.5 ± 33.5 mg/dL. Subsequent glimepiride treatment reduced these to 291.8 ± 48.5 mg∙h/dL and 134.4 ± 26.9 mg/dL, reflecting reductions of 67.5 ± 60 mg∙h/dL and 68.9 ± 25.1 mg/dL, respectively. Pertinent to this study’s supplementary data, the combined therapy of OJS and glimepiride showed a 50.81 ± 18.02 mg∙h/dL and 45.23 ± 14.54 mg/dL reduction of AUG_0–3 h_ and G_max_ compared to OJS monotherapy (Supplementary materials, Table S1). When comparing the mean and standard deviation of AUG and G_max_ reduction from baseline between the current and previous studies, it appears that the combined therapy of OJS and glimepiride has only a limited additional impact on glycemic control compared to glimepiride monotherapy.

In contrast to the findings of the present study, the previously reported favorable hypoglycemic effects of OJS were derived from retrospective analyses conducted in hospitalized patients with T2DM^27^. It is likely that strict dietary control and the consistent use of antidiabetic medications during hospitalization significantly influenced those outcomes. Therefore, in the absence of rigorously controlled prospective studies, the actual impact of OJS on glycemic control remains uncertain. To determine whether combination therapy with OJS and glimepiride produces clinically meaningful PD effects—warranting dosage or regimen adjustments—future prospective studies are required. Such studies should include patients undergoing long-term glimepiride therapy and assess relevant endpoints such as PP2 and HbA1c. Moreover, given the limited sample size of the current study, additional research is essential to comprehensively evaluate PD markers, including HbA1c, and confirm the consistency and clinical significance of the observed effects.

The study findings indicated that the co-administration of glimepiride and OJS was generally well-received by healthy male subjects. Notable side effects of glimepiride, such as dizziness and increased perspiration, have been reported in a subset of subjects. These adverse reactions were mild in intensity and resolved spontaneously before the study conclusion. Moreover, the concurrent intake of OJS with glimepiride resulted in decreased systemic exposure to glimepiride, accompanied by sweating among some male subjects. Based on these observations, it is plausible that the synergistic use of glimepiride and OJS is a viable and tolerable therapeutic strategy. Nevertheless, it is critical to underline the necessity for further investigations involving broader cohorts to confirm the sustained safety and efficacy of this combination therapy over the long term.

The present study has several limitations. The final PK analysis included 13 participants, fewer than initially planned. To assess the impact of this reduction, intrasubject variability was estimated using the 90% CI of the GMR for AUC, yielding a coefficient of variation of approximately 16%, indicating low variability. A previous glimepiride interaction study also reported reliable results with as few as 12 participants^40^. Notably, the present study successfully captured the reduction in C_max_ during the absorption phase when glimepiride was co-administered with OJS. Therefore, despite the limited sample size, the dataset is considered to provide meaningful PK results. Moreover, this study was conducted on healthy subjects to minimize confounding factors. However, in patients with diabetes mellitus, the PK and safety profiles may differ from the current study. At present, it is challenging to definitively conclude whether glimepiride dose modifications are necessary with OJS use, and more comprehensive studies are needed to provide conclusive evidence. Nevertheless, this study offers important insights into the PK interaction between glimepiride and OJS through quantitative evaluation in healthy volunteers.

## Methods

### Subject

Healthy male subjects 19 to 45 years of age with a body mass index in the range of 18.0 to 29.0 kg/m^2^ were enrolled in this study. Participants provided written informed consent after receiving a detailed explanation of the study and were screened for eligibility. The screening assessment included medical history, physical examination, 12-lead electrocardiography, and clinical laboratory tests. The required sample size to detect a ≥ 20% difference in key PK parameters from the interaction between glimepiride and OJS was 16 subjects (α = 0.05, power = 90%), with a final sample size of 18 accounting for a 10% dropout rate. The study protocol and informed consent forms were approved by the Korean Ministry of Food and Drug Safety, Institutional Review Board (Registration No. KHUH 2021-05-007) of Kyung Hee University Hospital (KHUH, Seoul, Republic of Korea) on 09/06/2021, and IRB (Registration No. KOMCIRB 2021-06-004) of Kyung Hee University Korean Medical Hospital (KHUKMH, Seoul, Republic of Korea) on 07/09/2021.

### Design

This was an open-label, single-sequence, crossover clinical study. The study was conducted in accordance with the Declaration of Helsinki and was registered in Korean Good Clinical Practice (Clinical Research Information Service, CRIS [https://cris.nih.go.kr]; registry number: KCT0006799) on 02/12/2021. The recruitment of patients started on (09/12/2021) and the trial ended at (21/12/2022). The present clinical trial is in accordance with CONSORT guidelines^41^.

The schedule of the current study comprised a screening visit, period 1, period 2, and post-study visit. All participants visited on one day during the screening period, from 28 days to 1 d (day − 1) before the first drug administration, and they were assessed for eligibility based on the study’s inclusion and exclusion criteria.

The inclusion criteria were as follows; (1) Healthy males aged 19–45 at screening, (2) Body weight: 50–90 kg; body mass index (BMI): 18.0–29.0 kg/m², (3) Voluntarily consented and agreed to comply with study procedures after full explanation, (4) Determined to be healthy by investigator based on screening assessments (physical exam, labs, interview, etc.). And the exclusion criteria were as follows; (1) History of significant disease (hepatic, gastrointestinal, cardiovascular, renal, respiratory, endocrine, neurologic, immunologic, hematologic, psychiatric), neoplasm, or drug abuse (2) Known hypersensitivity to the investigational product or related drugs (3) Contraindications to the investigational product (e.g., galactose intolerance, Lapp lactase deficiency, glucose-galactose malabsorption) (4) Gastrointestinal diseases (Crohn’s disease, ulcers, acute or chronic pancreatitis, etc.) or surgeries (except appendectomy or hernia repair) affecting drug absorption (5) Abnormal clinical findings at screening: 5 − 1) Systolic blood pressure (SBP) < 90 or > 150 mmHg, Diastolic blood pressure (DBP) < 60 or > 100 mmHg, 5 − 2) Aspartate aminotransferase (AST) / Alanine aminotransferase (ALT) > 1.5× upper limit of normal (ULN), 5 − 3) Estimated glomerular filtration rate (eGFR) < 60 mL/min/1.73 m² (Modification of Diet in Renal Disease (MDRD) formula), 5 − 4) Positive or abnormal serology (hepatitis B surface antigen (HBsAg), anti-hepatitis C virus antibody (anti-HCV), human immunodeficiency virus antibody (HIV Ab), rapid plasma reagin (RPR)) (6) Use of restricted substances prior to dosing: 6 − 1) CYP enzyme inducers/inhibitors within 4 weeks, 6 − 2) Prescription meds or herbal remedies within 2 weeks; 6 − 3) OTC drugs within 1 week, 6 − 4) Enzyme-affecting drugs (e.g., barbiturates) within 1 month or interfering drugs within 10 days 6 − 5) Grapefruit or its products within 72 h, 6–6) Caffeine or alcohol within 72 h (or chronic alcohol use within 6 months), (7) Participation in other clinical trials within 6 months, (8) Inadequate contraception or plans for pregnancy (self/partner) from consent to 2 weeks post-study, (9) Whole blood donation within 2 months, apheresis/blood transfusion within 1 month, (10) Inability to abstain from excessive physical activity from 72 h pre-dose to study end, (11) Current smoker or positive cotinine test, (12) Noncompliance with study procedures or investigator’s judgment, (13) Inability to read or understand the consent form, (14) Any condition deemed inappropriate by the investigator.

**Fig. 3:**
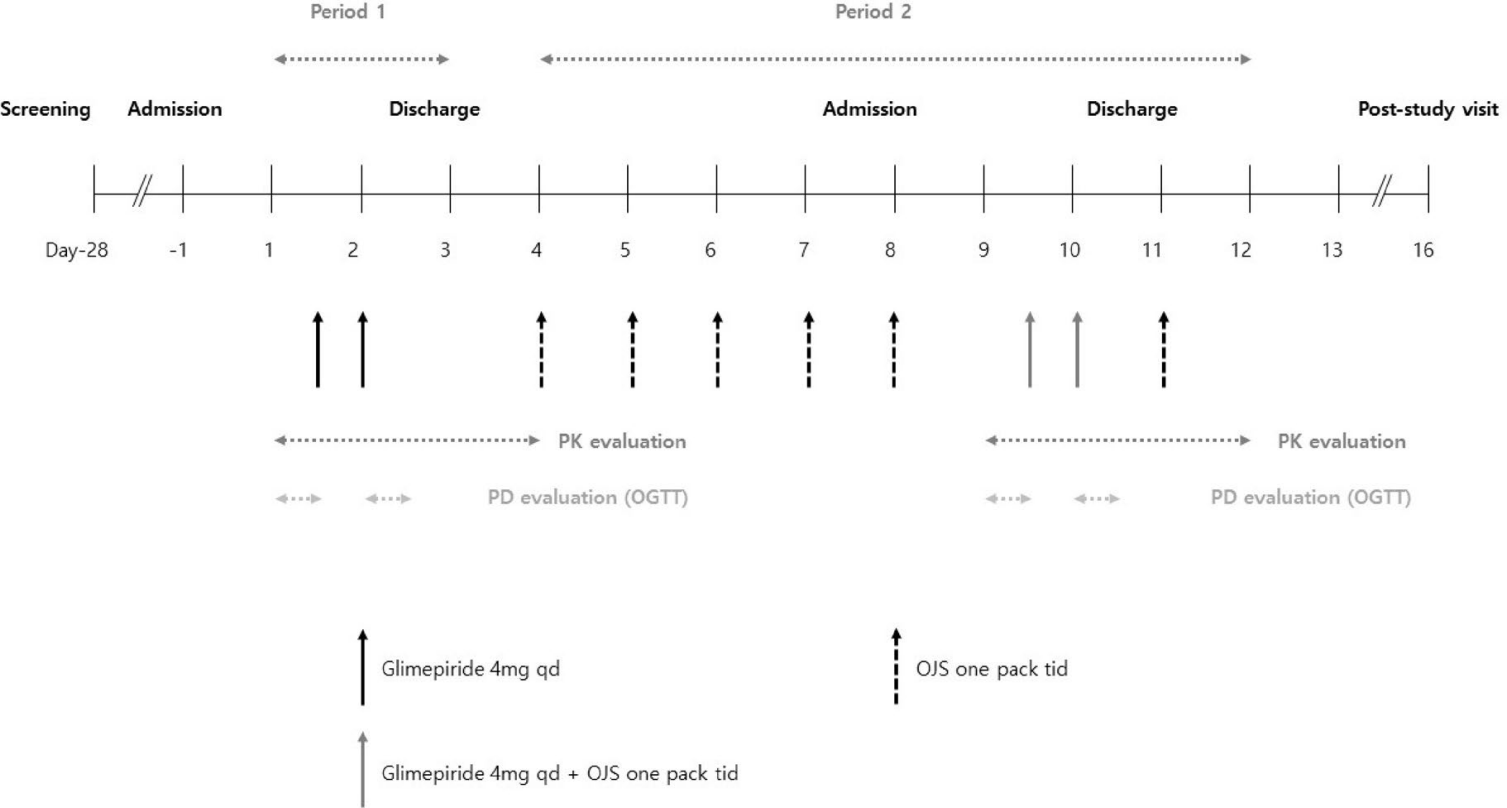
Treatment schema for dosing schedules of glimepiride and OJS. The days on which glimepiride and/or OJS were administered are denoted with arrows. Additionally, the schedule for blood sampling for pharmacokinetic and pharmacodynamic analysis is depicted. PK, Pharmacokinetic; PD, Pharmacodynamic; OGTT, oral glucose tolerance test; OJS, Ojeok-san; qd, once daily; tid, three times daily.

During period 1 (days 1–3), all participants were hospitalized on day − 1 and discharged on day 3. In period 2 (days 4–12), patients were hospitalized on day 8 and discharged on day 11. All subjects were provided standard meals during hospitalization and were required to fast for 8 h before the oral glucose tolerance test (OGTT). During the post-study visit, they finally visited the clinical trial center for safety evaluation before the end of the study (Fig. 3).

Glimepiride (4 mg) was orally administered once daily on days 1–2 during period 1. Subsequently, the 4.35 g/pack of OJS was orally administered three times a day on days 4–11 during period 2, and glimepiride (4 mg) was co-administered once a day on days 9–10 during period 2. Glimepiride (4 mg) was administered at approximately 12:00 PM on days 1 and 9 and at approximately 8:00 AM on days 2 and 10. The 4.35 g/pack of OJS was administered thrice a day at 7:00–10:00 AM, 12:00–03:00 PM, and 6:00–9:00 PM on days 4–11. However, during hospitalization, OJS was administered at approximately 7:00 AM on days 9–10, just 1 h before glimepiride administration.

For the PK analysis of glimepiride, blood samples were collected immediately before the glimepiride administration on days 1 and 9 on the first day of each period. On days 2 and 10, blood samples were collected immediately before glimepiride administration (0 h) and 0.25, 0.5, 1, 1.5, 2, 2.5, 3, 3.5, 4, 5, 6, 8, 12, 15, 24, and 48 h after glimepiride administration. Oral glucose tolerance tests (OGTT) were performed before and after glimepiride administration on days 1 and 9, and days 2 and 10, respectively, to assess the PD effects (Supplementary information).

### Determination of glimepiride concentration

Plasma glimepiride and hydroxy glimepiride concentrations were estimated by LC–MS/MS using an API 4000 triple quadrupole mass spectrometer (AB SCIEX, Framingham, Massachusetts, U.S.A.) coupled to an Agilent 1200 series HPLC system (Agilent Technologies, Waldronn, Germany). The analyte was separated using a YMC hydrosphere C18 (50 × 2.0 mm, 3 μm; YMC, USA) at 40 ℃. The mobile phase consists of 5 mM ammonium formate and 100% acetonitrile (20:80, v/v) with a flow rate of 180 µL/min. The compounds were detected in the positive mode using an electrospray ionization source. The mass spectrometer was operated in multiple reaction monitoring mode with a mass-to-charge ratio (m/z) of 491.30 → 352.10 for glimepiride and 507.38 → 352.10 for hydroxy glimepiride. A linear response of glimepiride was shown in the range of 2 to 1000 ng/mL (*r*^2^ ≥ 0.9970), and the lower limit of quantification was determined as 2 ng/mL. A linear response of hydroxy glimepiride was shown in the range of 0.5 to 100 ng/mL (*r*^2^ ≥ 0.9980), and the lower limit of quantification (LLOQ) was determined as 0.5 ng/mL. The intra- and inter-day precisions for glimepiride were less than 6.36% and 5.79%, respectively, whereas those for hydroxy glimepiride were less than 14.01% and 15.99%, respectively. The intra- and inter-day accuracies for glimepiride and hydroxy glimepiride were less than 5.37 and 3.03% and less than 15.11 and 5.59%, respectively.

### Pharmacokinetic analysis

PK parameters were calculated using the noncompartmental method in Phoenix WinNonlin 8.3 (Certara USA Inc., Princeton, NJ, USA). The PK parameters for glimepiride and hydroxy glimepiride (M1) were calculated as follows: The maximum plasma concentration (C_max_) and time to reach C_max_ (T_max_) were directly determined using the observed individual time-concentration profiles. The terminal elimination rate constant (kz) was estimated from the regression of the log-linear decrease in the plasma concentration-time profile. The terminal elimination half-life (t_1/2_) was calculated as the natural logarithm of 2 divided by kz. The area under the plasma concentration-time curve (AUC) from dosing to 24 h post-dosing (AUC_0–24 h_) and AUC_0–48 h_ from dosing to 48 h post-dosing were calculated using the linear/log trapezoidal rule. The AUC from 0 to infinity (AUC_inf_) was calculated as AUC_0–__24 h_ + C_24_ / kz, where kz is the terminal elimination rate constant estimated from linear regression of the terminal phase of the log-linear plot of the plasma concentration-time curve. The metabolic ratio was calculated by dividing the M1 AUC_0–24 h_ by glimepiride AUC_0–24 h_.

### Supplementary pharmacodynamic findings

The PD results are reported in the supplementary material, due to lapse in protocol adherence. The PD parameters included the maximum serum glucose concentration (G_max_), 2-hour serum glucose level (PP2), and area under the glucose concentration-time curve for 4 h after glucose administration (AUG_0–4 h_), which was calculated using the linear trapezoidal rule. The change values of G_max_, PP2, and AUG_0–4 h_ were calculated by subtracting the baseline serum glucose concentrations from the corresponding post-administration values at each time point, following glimepiride administration with or without OJS. The maximum of these difference values was defined as the change in G_max_, and the value at 2 h as the change in PP2. The area under the curve from 0 to 4 h was calculated using linear trapezoidal method and defined as change in AUG_0 − 4 h_. Additionally, the percent change (%change) was calculated as: 100×(change value / baseline value).

### Genotype analysis

Single nucleotide polymorphisms (SNP) in CYP2C9 were genotyped to identify CYP2C9*3 (rs1057910). Genotyping for SNP was performed by TaqMan assay using Exicycler™ 96 Real-Time PCR System (Bioneer, South Korea). Primer and probe for the genotyping were synthesized by Bioneer Co., and two dyes, FAM and Cy5 were used in fluorescent probes.

### Safety and tolerability assessment

Safety and tolerability were assessed during the study period. The occurrence of AEs was monitored, and the investigators assessed its causal relationship with the study drugs. Vital signs, 12-lead electrocardiography (ECGs), physical examinations, and laboratory tests were performed and reviewed. Changes from baseline with respect to these results were considered in the tolerability assessment.

### Statistical analysis

All statistical analyses were performed using SAS 9.4 (SAS Institute Inc. Cary, NC, USA). Descriptive statistics were calculated for continuous variables. C_max_ and AUC were naturally log-transformed and compared using a paired t-test between the co-administration of glimepiride and OJS and glimepiride alone. Mean differences with 90% confidence intervals (CI) for C_max_ and AUC were exponentiated (anti-Ln transformed) to obtain the Geometric Mean Ratios (GMR) with 90% CI. These 90% CIs were compared with the bioequivalence range of 80.00 to 125.00 to quantify the extent of herb-drug interactions. The glucose-lowering effect, change, or % change in AUG_0–4 h_, G_max_ and PP2 levels were tested using a paired t-test. All figures used the ggplot2 package of the R program^42^.

## Electronic supplementary material

Below is the link to the electronic supplementary material.

Supplementary Material 1

## Data Availability

The data supporting the results of this study are available from the corresponding author upon reasonable request.

## References

[1] Sun, H. et al. IDF diabetes atlas: global, regional and country-level diabetes prevalence estimates for 2021 and projections for 2045. *Diabetes Res. Clin. Pract.* **183**, 109119 (2022). 34879977 10.1016/j.diabres.2021.109119 PMC11057359

[2] Zhou, B. et al. Worldwide trends in diabetes since 1980: A pooled analysis of 751 population-based studies with 4· 4 million participants. *Lancet* **387**, 1513–1530 (2016). 27061677 10.1016/S0140-6736(16)00618-8 PMC5081106

[3] Basit, A., Riaz, M. & Fawwad, A. Glimepiride: evidence-based facts, trends, and observations (GIFTS). [corrected]. *Vasc Health Risk Manag*. **8**, 463–472 (2012). 23028231 10.2147/HIV.S33194 PMC3448454

[4] UK Prospective Diabetes Study (UKPDS) Group. Intensive blood-glucose control with sulphonylureas or insulin compared with conventional treatment and risk of complications in patients with type 2 diabetes (UKPDS 33). *Lancet* **352**, 837–853 (1998). 9742976

[5] Scheen, A. J. Sulphonylureas in the management of type 2 diabetes: to be or not to be? *Diabetes Epidemiol. Manage.* **1**, 100002 (2021).

[6] Engler, C. et al. Long-term trends in the prescription of antidiabetic drugs: real-world evidence from the diabetes registry Tyrol 2012–2018. *BMJ Open. Diabetes Res. Care*. **8**, e001279 (2020). 32873600 10.1136/bmjdrc-2020-001279 PMC7467522

[7] Shamanna, P. et al. Multicenter, retrospective, study on the usage patterns of the fixed dose combination of glimepiride, metformin, and Voglibose in type 2 diabetes management. *Cureus* **16**, e52064 (2024). 38348001 10.7759/cureus.52064 PMC10859676

[8] Mohan, V. et al. Position of sulfonylureas in the current ERA: review of National and international guidelines. *Clin. Med. Insights Endocrinol. Diabetes*. **15**, 11795514221074663 (2022). 35185350 10.1177/11795514221074663 PMC8854230

[9] Klatt, S., Fromm, M. F. & König, J. Transporter-mediated drug–drug interactions with oral antidiabetic drugs. *Pharmaceutics* **3**, 680–705 (2011). 24309303 10.3390/pharmaceutics3040680 PMC3857053

[10] Chen, Y. et al. Interaction of sulfonylureas with liver uptake transporters OATP1B1 and OATP1B3. *Basic. Clin. Pharmacol. Toxicol.* **123**, 147–154 (2018). 29498478 10.1111/bcpt.12992

[11] Vaidyanathan, J., Choe, S. & Sahajwalla, C. G. Type 2 diabetes in pediatrics and adults: thoughts from a clinical Pharmacology perspective. *J. Pharm. Sci.* **101**, 1659–1671 (2012). 22383396 10.1002/jps.23085

[12] Niemi, M., Kivistö, K. T., Backman, J. T. & Neuvonen, P. J. Effect of rifampicin on the pharmacokinetics and pharmacodynamics of glimepiride. *Br. J. Clin. Pharmacol.* **50**, 591–595 (2000). 11136298 10.1046/j.1365-2125.2000.00295.x PMC2015006

[13] Niemi, M. et al. Effects of fluconazole and fluvoxamine on the pharmacokinetics and pharmacodynamics of glimepiride. *Clin. Pharmacol. Ther.* **69**, 194–200 (2001). 11309547 10.1067/mcp.2001.114229

[14] Mamun, A. A. & Khan, M. S. S. A review of significance of herbal medicine and its evolution as a therapeutics in global healthcare. *Australian Herb. Insight*. **3**, 1–9 (2020).

[15] Xue, C. C., Zhang, A. L., Lin, V., Da Costa, C. & Story, D. F. Complementary and alternative medicine use in Australia: a national population-based study. *J. Altern. Complement. Med.* **13**, 643–650 (2007). 17718647 10.1089/acm.2006.6355

[16] Evans, A., Duncan, B., McHugh, P., Shaw, J. & Wilson, C. Inpatients’ use, understanding, and attitudes towards traditional, complementary and alternative therapies at a provincial new Zealand hospital. *N. Z. Med. J.* **121**, 21–34 (2008). 18670472

[17] Yea, S., Jang, H., Kim, S., Lee, S. & Kim, J. U. Annotated corpus for traditional formula-disease relationships in biomedical articles. *Sci. Data* **12**, 26 (2025). 39774689 10.1038/s41597-025-04377-2 PMC11707285

[18] Ko, Y. et al. Efficacy and safety of Ojeok-san in Korean female patients with cold hypersensitivity in the hands and feet: study protocol for a randomized, double-blinded, placebo-controlled, multicenter pilot study. *Trials* **19**, 662 (2018). 30497488 10.1186/s13063-018-3013-9 PMC6267068

[19] Kim, J. H., Seo, C. S., Kim, S. S. & Shin, H. K. Quality assessment of ojeok-san, a traditional herbal formula, using high-performance liquid chromatography combined with chemometric analysis. *J. Anal. Methods Chem.* **2015**, 607252 (2015). 10.1155/2015/607252 PMC4619932 26539304

[20] Lyu, Y. R. et al. Efficacy and safety of Ojeok-San plus Saengmaek-San for gastroesophageal Reflux-Induced chronic cough: A pilot, randomized, Double-Blind, Placebo-Controlled trial. *Front. Pharmacol.* **13**, 787860 (2022). 35300295 10.3389/fphar.2022.787860 PMC8923584

[21] National Health Insurance Service (NHIS). Report on insurance claims for reimbursement from the National Health Insurance Service (NHIS). https://www.hira.or.kr/bbsDummy.do?pgmid=HIRAA020045010000&brdScnBltNo=4&brdBltNo=2405&pageIndex=1&pageIndex2=1 (2023).

[22] Shahrzad, S., Aoyagi, K., Winter, A., Koyama, A. & Bitsch, I. Pharmacokinetics of Gallic acid and its relative bioavailability from tea in healthy humans. *J. Nutr.* **131**, 1207–1210 (2001). 11285327 10.1093/jn/131.4.1207

[23] Heo, S. H. et al. Pharmacokinetic evaluation of Paeoniflorin after oral administration of paeoniae Radix extract powder to healthy Korean subjects using UPLC-MS/MS. *J. Pharm. Invest.* **46**, 273–282 (2016).

[24] Yang, C., Tian, Y., Zhang, Z., Xu, F. & Chen, Y. High-performance liquid chromatography-electrospray ionization mass spectrometry determination of sodium ferulate in human plasma. *J. Pharm. Biomed. Anal.* **43**, 945–950 (2007). 17049197 10.1016/j.jpba.2006.09.027

[25] Dominguez-Avila, J. A. et al. Gastrointestinal interactions, absorption, splanchnic metabolism and pharmacokinetics of orally ingested phenolic compounds. *Food Funct.* **8**, 15–38 (2017). 28074953 10.1039/c6fo01475e

[26] Brantley, S. J., Argikar, A. A., Lin, Y. S., Nagar, S. & Paine, M. F. Herb-drug interactions: challenges and opportunities for improved predictions. *Drug Metab. Dispos.* **42**, 301–317 (2014). 24335390 10.1124/dmd.113.055236 PMC3935140

[27] Lee, M. S. et al. A retrospective study on the effect of the co-administration of Ojeok-san and hypoglycemic agents on blood glucose levels in type 2 diabetes mellitus. *J. Int. Korean Med.* **42**, 40–52 (2021).

[28] Yoo, H., Kim, Y., Jang, I. J., Yu, K. S. & Lee, S. Pharmacokinetic/Pharmacodynamic interactions between evogliptin and glimepiride in healthy male subjects. *Drug Des. Devel*.* Ther.* **14**, 5179–5187 (2020). 33262578 10.2147/DDDT.S275343 PMC7699451

[29] Azher, M. et al. The clinical pharmacokinetics and pharmacodynamics of glimepiride-a systematic review and meta-analysis. *Pharmaceuticals (Basel)*. **18**, 122 (2025). 39861183 10.3390/ph18010122 PMC11768776

[30] Park, S. I., Park, J. Y., Park, M. J., Yim, S. V. & Kim, B. H. Effects of Ojeok-san on the pharmacokinetics of celecoxib at Steady-state in healthy volunteers. *Basic. Clin. Pharmacol. Toxicol.* **123**, 51–57 (2018). 29377603 10.1111/bcpt.12971

[31] Sanofi-Aventics. *Amaryl® (glimepiride) prescribing information* (Sanofi-Aventis, 2018).

[32] He, L. et al. The effects of colesevelam HCl on the single-dose pharmacokinetics of glimepiride, extended-release glipizide, and Olmesartan Medoxomil. *J. Clin. Pharmacol.* **54**, 61–69 (2014). 24019110 10.1002/jcph.180

[33] Kimura, Y., Ito, H., Ohnishi, R. & Hatano, T. Inhibitory effects of polyphenols on human cytochrome P450 3A4 and 2C9 activity. *Food Chem. Toxicol.* **48**, 429–435 (2010). 19883715 10.1016/j.fct.2009.10.041

[34] Jin, S. E., Seo, C. S., Shin, H. K. & Ha, H. Traditional herbal formulas to as treatments for musculoskeletal disorders: their inhibitory effects on the activities of human microsomal cytochrome P450s and UDP-glucuronosyltransferases. *Pharmacogn. Mag*. **12**, 241–252 (2016). 27867264 10.4103/0973-1296.192205 PMC5096268

[35] Kim, K. A., Song, W. G., Lee, H. M., Joo, H. J. & Park, J. Y. Multiplex pyrosequencing method to determine CYP2C9*3, VKORC1*2, and CYP4F2*3 polymorphisms simultaneously: its application to a Korean population and comparisons with other ethnic groups. *Mol. Biol. Rep.* **41**, 7305–7312 (2014). 25069408 10.1007/s11033-014-3617-4

[36] Bae, J. W. et al. Frequency of CYP2C9 alleles in Koreans and their effects on Losartan pharmacokinetics. *Acta Pharmacol. Sin*. **32**, 1303–1308 (2011). 21841812 10.1038/aps.2011.100 PMC4010224

[37] Miron, A., Aprotosoaie, A. C., Trifan, A. & Xiao, J. Flavonoids as modulators of metabolic enzymes and drug transporters. *Ann. N. Y. Acad. Sci.* **1398**, 152–167 (2017). 28632894 10.1111/nyas.13384

[38] Liu, M. et al. Berberine Promotes OATP1B1 Expression and Rosuvastatin Uptake by Inducing Nuclear Translocation of FXR and LXRα. *Front. Pharmacol.* **11**, 375 (2020). 32292349 10.3389/fphar.2020.00375 PMC7118773

[39] Hwang, I. et al. Pharmacokinetic/pharmacodynamic interaction between evogliptin and Pioglitazone in healthy male subjects. *Drug Des. Devel. Ther.* **14**, 4493–4502 (2020). 33122892 10.2147/DDDT.S275336 PMC7591087

[40] Que, L. et al. No apparent Pharmacokinetic interactions were found between henagliflozin: a novel sodium-glucose co-transporter 2 inhibitor and glimepiride in healthy Chinese male subjects. *J. Clin. Pharm. Ther.* **47**, 1225–1231 (2019). 10.1111/jcpt.13659 35362180

[41] Dwan, K., Li, T., Altman, D. G. & Elbourne, D. CONSORT 2010 statement: extension to randomised crossover trials. *BMJ* **366**, l4378 (2019). 31366597 10.1136/bmj.l4378 PMC6667942

[42] Wickham, H. ggplot2: Elegant graphics for data analysis. (Springer, 2016).

