# Supplementary material for: Comparisons of pharmacokinetics of glimepiride in combination with Ojeok-san versus glimepiride alone: an open-label, one-sequence, two-treatment controlled clinical study
Source: Sci Rep. 2025 Jul 16;15:25813. doi: 10.1038/s41598-025-09317-z (PMC12267423; doi:10.1038/s41598-025-09317-z)
Supplement: Supplementary file 1 — Supplementary Material 1 [file 41598_2025_9317_MOESM1_ESM.docx]

# Comparisons of Pharmacokinetics of Glimepiride in Combination with Ojeok-san versus Glimepiride Alone: An Open-Label, One-Sequence, Two-Treatment Controlled Clinical Study

Jongyoon Kim^1^

Minji Kwon^2^

Sooyoung Lee^2,3^

Jeein Noh^4^

Wang-Seob Shim^5^

Eunseo Song^6^

Kyung-Tae Lee^5,6,7^

Ji-Young Park^8^

Sung-Vin Yim^2,9^

Bo-Hyung Kim^2,3,9,10^

^1^Department of Medicine, Graduate School, Kyung Hee University, Seoul, 02447, Republic of Korea; ^2^Department of Clinical Pharmacology and Therapeutics, Kyung Hee University Hospital, Seoul, 02447, Republic of Korea; ^3^East-West Medical Research Institute, Kyung Hee University, Seoul, 02447, Republic of Korea; ^4^Department of Regulatory Science, Graduate School, Kyung Hee University, Seoul, 02447, Republic of Korea; ^5^Kyung Hee Drug Analysis Center, College of Pharmacy, Kyung Hee University, Seoul, 02447, Republic of Korea; ^6^Department of Biomedical and Pharmaceutical Sciences, Graduate School, Kyung Hee University, Seoul, 02447, Republic of Korea; ^7^Department of Pharmaceutical Biochemistry, College of Pharmacy, Kyung Hee University, Seoul, 02447, Republic of Korea; ^8^Department of Clinical Pharmacology and Toxicology, Anam Hospital, Korea University College of Medicine, Seoul, 02841, Republic of Korea; ^9^Department of Clinical Pharmacology and Therapeutics, College of Medicine, Kyung Hee University, Seoul, 02447, Republic of Korea; ^10^Department of Biomedical Science and Technology, Graduate School, Kyung Hee University, Seoul, 02447, Republic of Korea

**Corresponding Author:** Bo-Hyung Kim; [bhkim98@khu.ac.kr](mailto:bhkim98@khu.ac.kr)

# Supplementary materials

The initial protocol stipulated the administration of 75 g of glucose; however, due to a lapse in protocol adherence by the administering clinical research nurse, which involved an incorrect glucose dosage of 50 g stemming from a failure to accurately verify the medication label, a deviation occurred (a report of this protocol violation was filed with the Institutional Review Board). Consequently, the plasma glucose assessment post-glimepiride monotherapy was conducted using 50 g of glucose, whereas the evaluation of the combined effect of glimepiride and OJS during the second admission utilized 75 g of glucose, introducing variance in glucose exposure across the two evaluations.

## Pharmacodynamics

Supplementary figure S2 illustrates the glycemic profiles following the administration of glimepiride as monotherapy and in combination with OJS, and figure S3 illustrates mean difference of glucose concentration-time curves between glimepiride alone and in combination with OJS. Comparative analysis revealed no statistically significant differences in AUG_0–4h_, AUG_0–3h_, G_max_, or PP2 between the administration of glimepiride alone and glimepiride co-administration with OJS (Supplementary Table S1). Supplementary figure S4 illustrates individual values of AUG_0–4h_, G_max_, and PP2 according to the time (Day 1, 2, 9, 10). The differences in glucose concentration and % change in PD parameters between glimepiride monotherapy and OJS combination treatment are illustrated in Supplementary figure S5 and S6.

**Supplementary Table S1.** Comparison of Pre-and Post-Administration of Oral Glucose Tolerance Test Results Between Glimepiride Monotherapy and Combination Therapy with Ojeok-san

|  | Glimepiride + OJS (N=13) | | Glimepiride (N=16) | |  |
| --- | --- | --- | --- | --- | --- |
|  | Pre-^a^ | Post- | Pre- | Post- | *P*-value^b^ |
| AUG_0–4 h_ (mg∙h /dL) | 489.19 ± 55.40 | 444.06 ± 61.94 | 466.93 ± 60.19 | 401.49 ± 42.34 |  |
| Change (mg∙h /dL) ^c^ | 70.08 ± 26.47 | | 77.01 ± 32.47 | | 0.6247 |
| % Change (%) | 14.32 ± 4.84 | | 16.15 ± 5.07 | | 0.3769 |
| AUG_0–3h_ (mg∙h /dL) | 401.48 ± 45.36 | 374.42 ± 51.83 | 383.74 ± 58.23 | 334.12 ± 44.24 |  |
| Change (mg∙h /dL) ^c^ | 50.81 ± 18.02 | | 61.07 ± 29.20 | | 0.3582 |
| % Change (%) | 12.75 ± 4.39 | | 15.54 ± 5.82 | | 0.1811 |
| G_max_ (mg/dL) | 178.23 ± 17.87 | 180.00 ± 17.81 | 178.75 ± 28.38 | 173.88 ± 20.38 |  |
| Change (mg/dL) | 45.23 ± 14.54 | | 45.63 ± 16.39 | | 0.9424 |
| % Change (%) | 25.93 ± 9.73 | | 25.48 ± 8.23 | | 0.8820 |
| PP2 (mg/dL) | 136.69 ± 25.51 | 119.62 ± 29.13 | 111.31 ± 29.13 | 95.75 ± 29.96 |  |
| Change (mg/dL) ^c^ | 23.08 ± 18.83 | | 17.94 ± 13.67 | | 0.2991 |
| % Change (%) | 16.63 ± 13.09 | | 16.88 ± 14.00 | | 0.9940 |

OJS, Ojeok-san; AUG_0–4 h_, area under the glucose concentration-time curve for 4 h after glucose administration; AUG_0_–_3 h_, the area under the glucose concentration-time curve 3 h after glucose administration; G_max_, maximum serum glucose concentration; PP2, 2-hour serum glucose. ^a^At “Pre”: oral glucose tolerance test was performed after the administration of OJS and before the administration of glimepiride. ^b^Paired t-test.


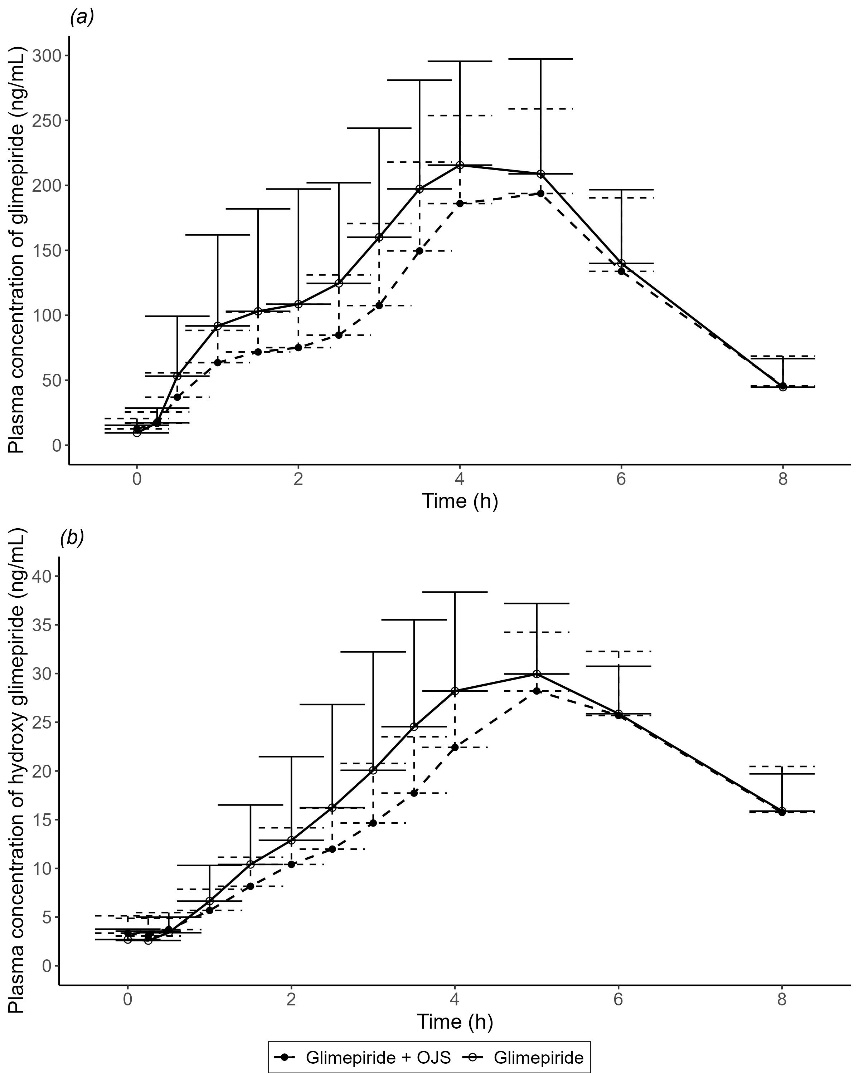


**Supplementary Fig. S1.** Mean plasma concentration-time curves from 0 to 8 hours for (a) glimepiride and (b) hydroxy glimepiride following monotherapy and combination therapy with OJS. Error bars indicate the standard deviations. OJS: Ojeok-san.


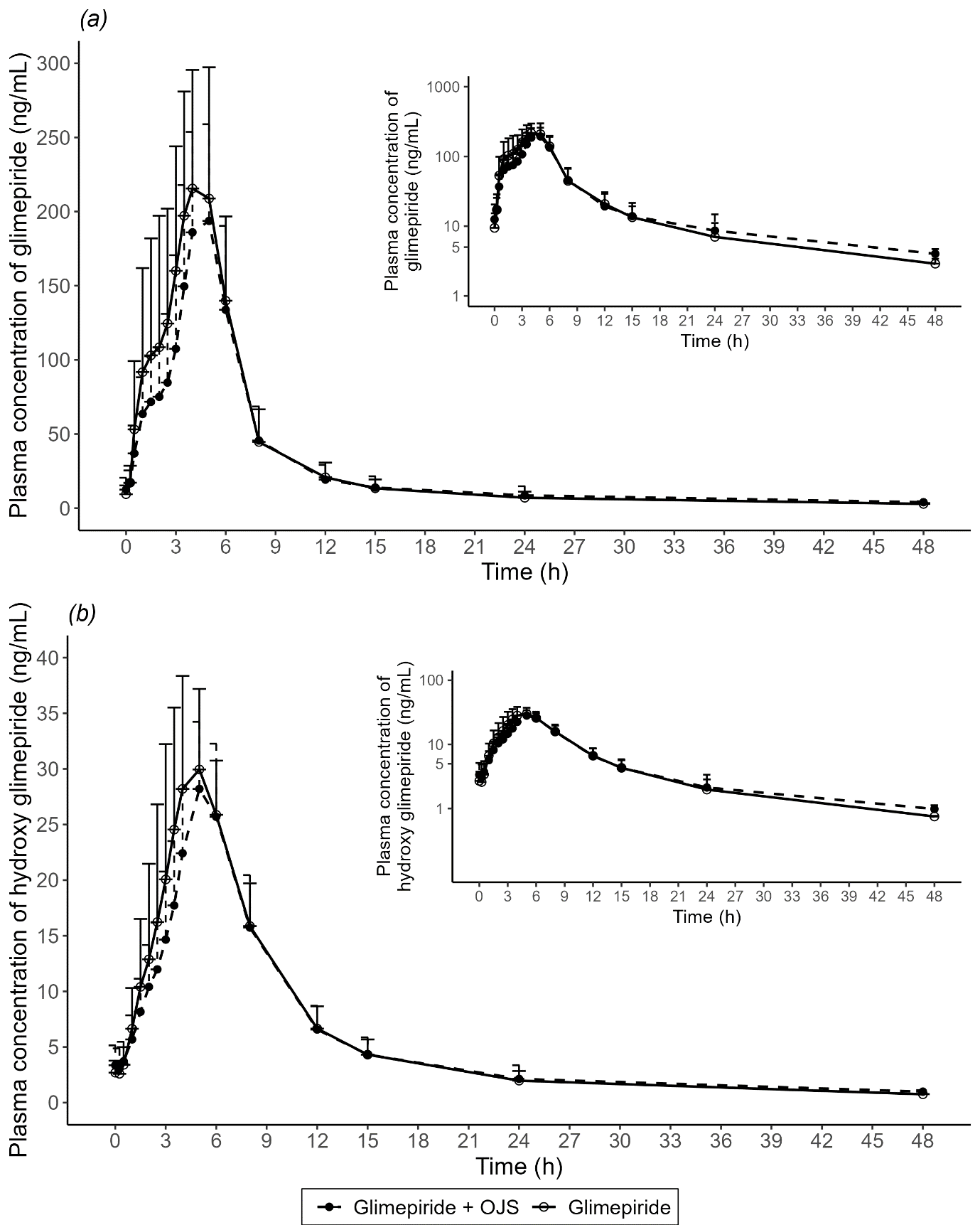


**Supplementary Fig. S2.** Mean plasma concentration-time curves from 0 to 48 hours for (a) glimepiride and (b) hydroxy glimepiride following monotherapy and combination therapy with OJS. Error bars indicate the standard deviations. OJS: Ojeok-san.


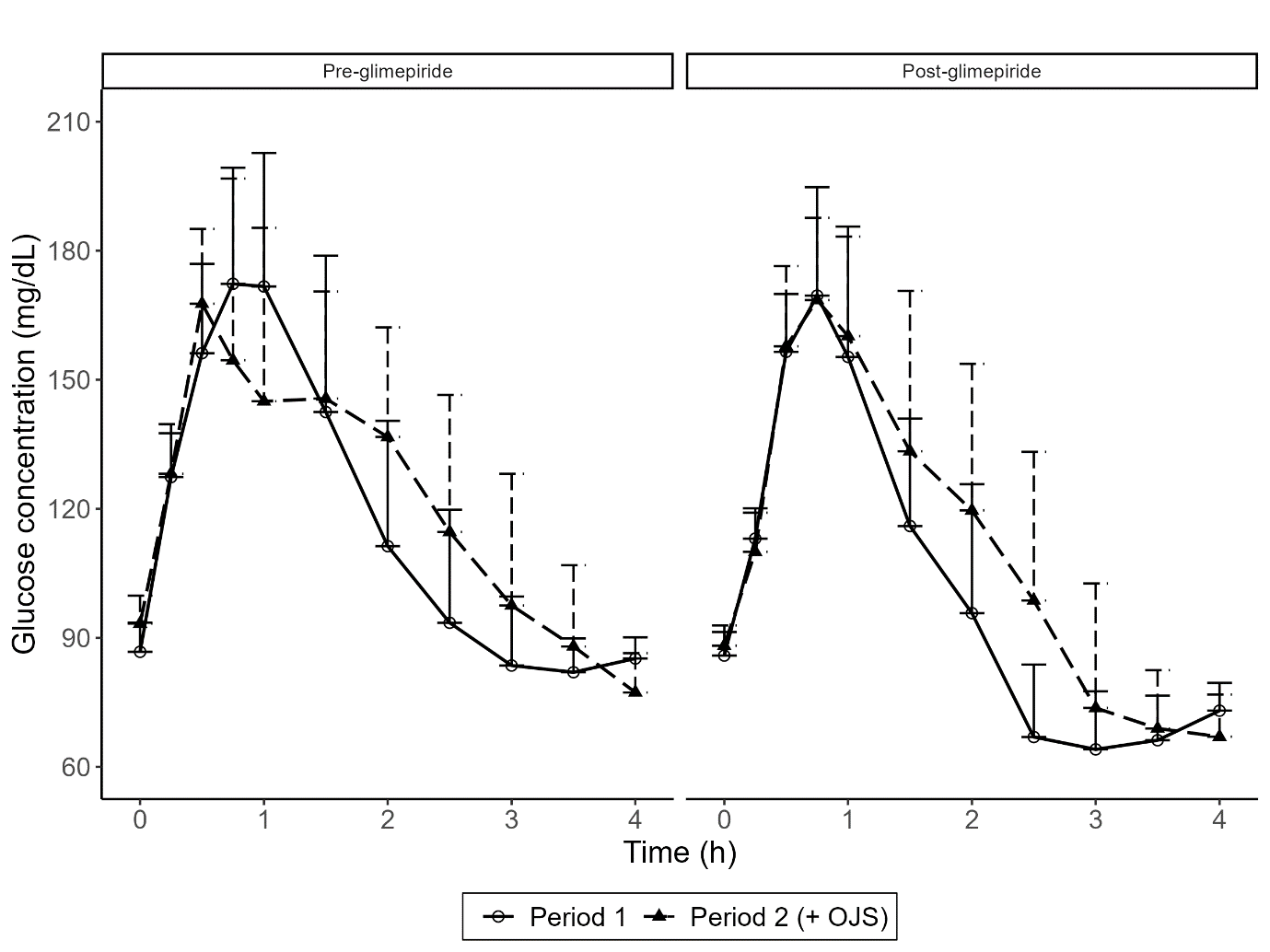


**Supplementary Fig. S3.** Mean serum glucose concentration-time profiles (a) pre-and (b) post-administration of glimepiride alone or in combination with OJS. Error bars indicate the standard deviations. OJS: Ojeok-san.


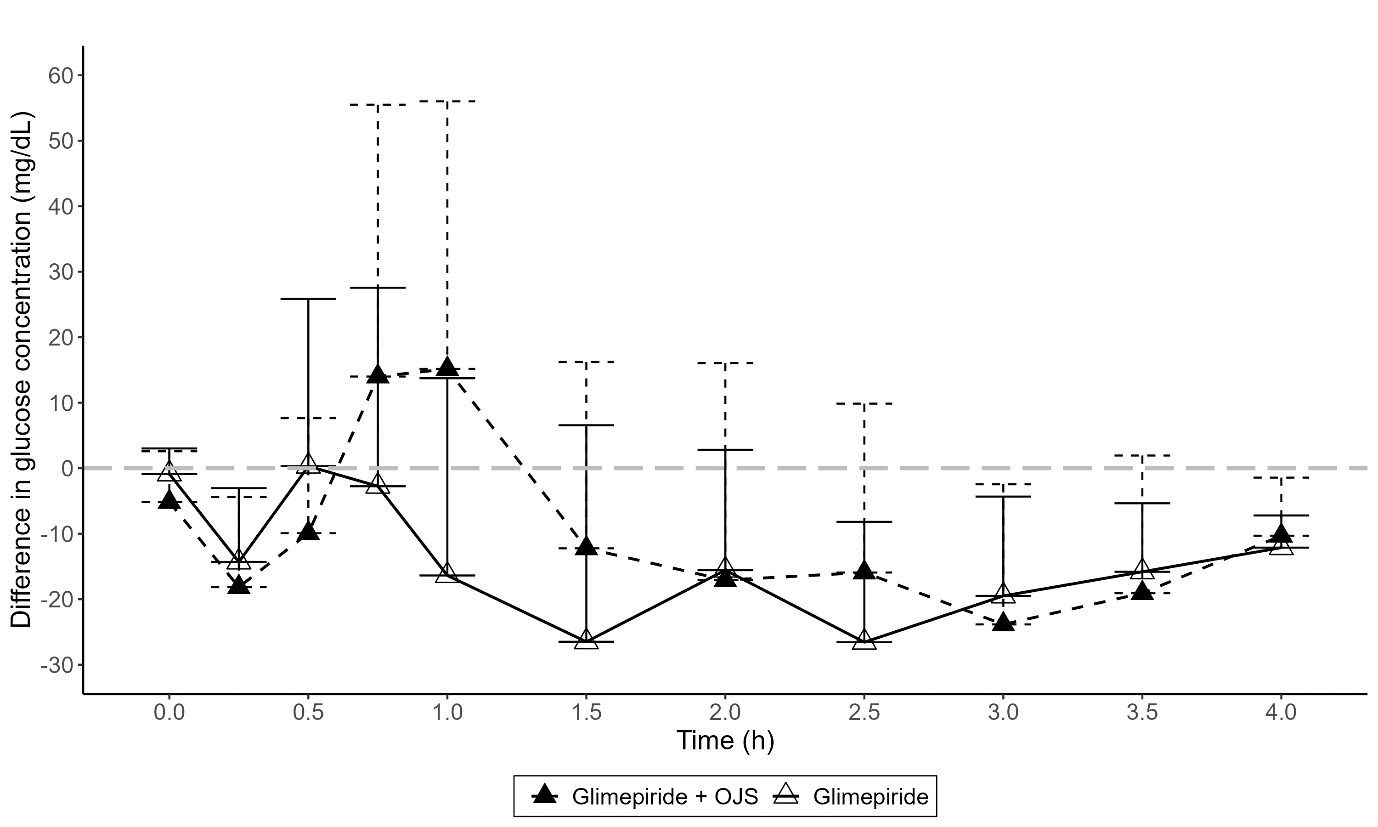


**Supplementary Fig. S4.** Mean difference of glucose concentration-time curves between glimepiride alone and in combination with OJS. Differences were calculated as “values at Post-Administration minus values at Pre-Administration” at each time point. Error bars indicate standard deviation. OJS: Ojeok-san.


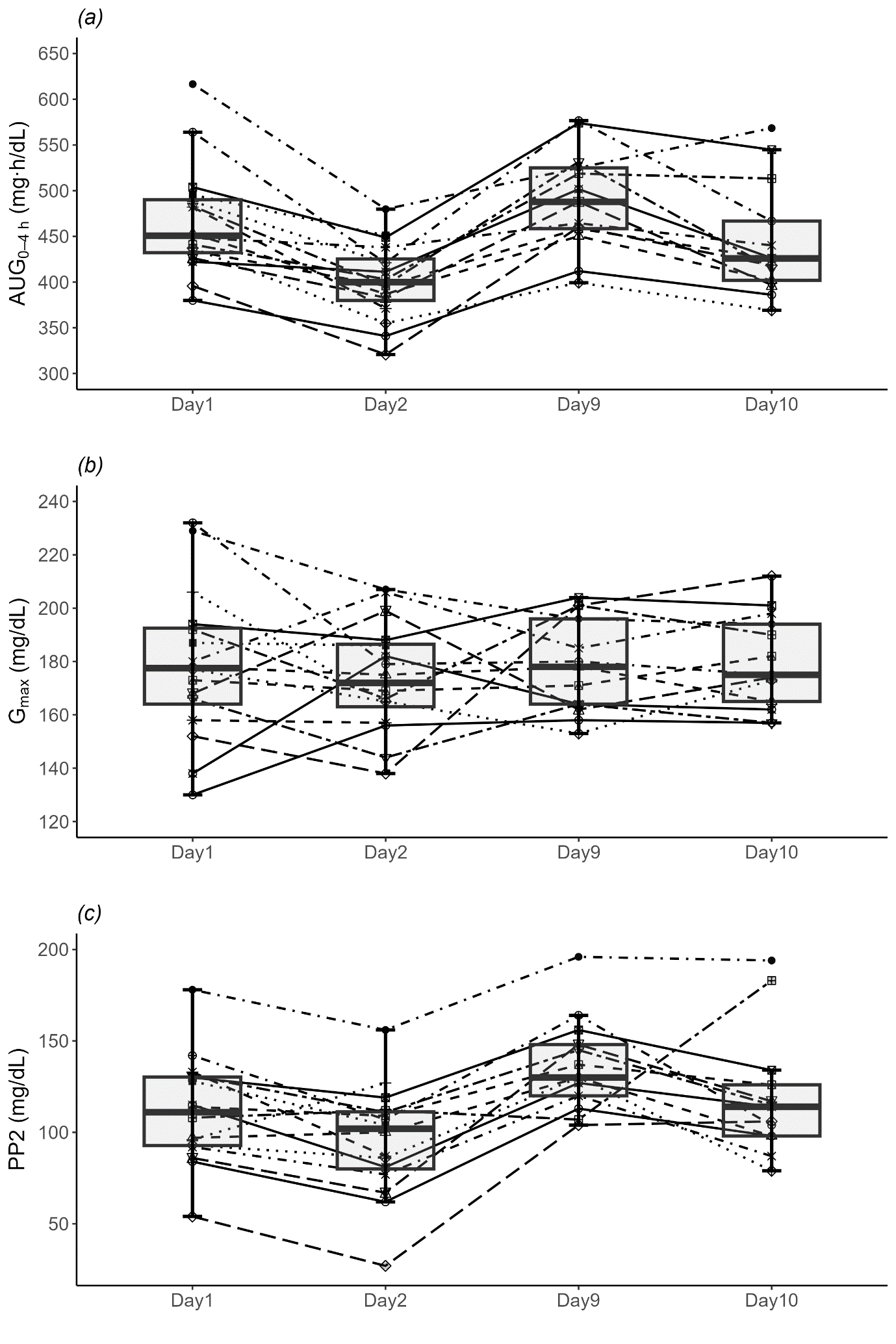


**Supplementary Fig. S5.** Individual change-time (Day 1, 2, 9, 10) profiles in (a) AUG_0–4 h_, (b) G_max_, and (c) PP2 for glimepiride alone or in combination with OJS. Box plots represent the lower quartile, median, and upper quartile. OJS, Ojeok-san; AUG_0–4 h_, area under the glucose concentration-time curve for 4 h after glucose administration; G_max_, maximum serum glucose concentration; PP2, 2-hour serum glucose.


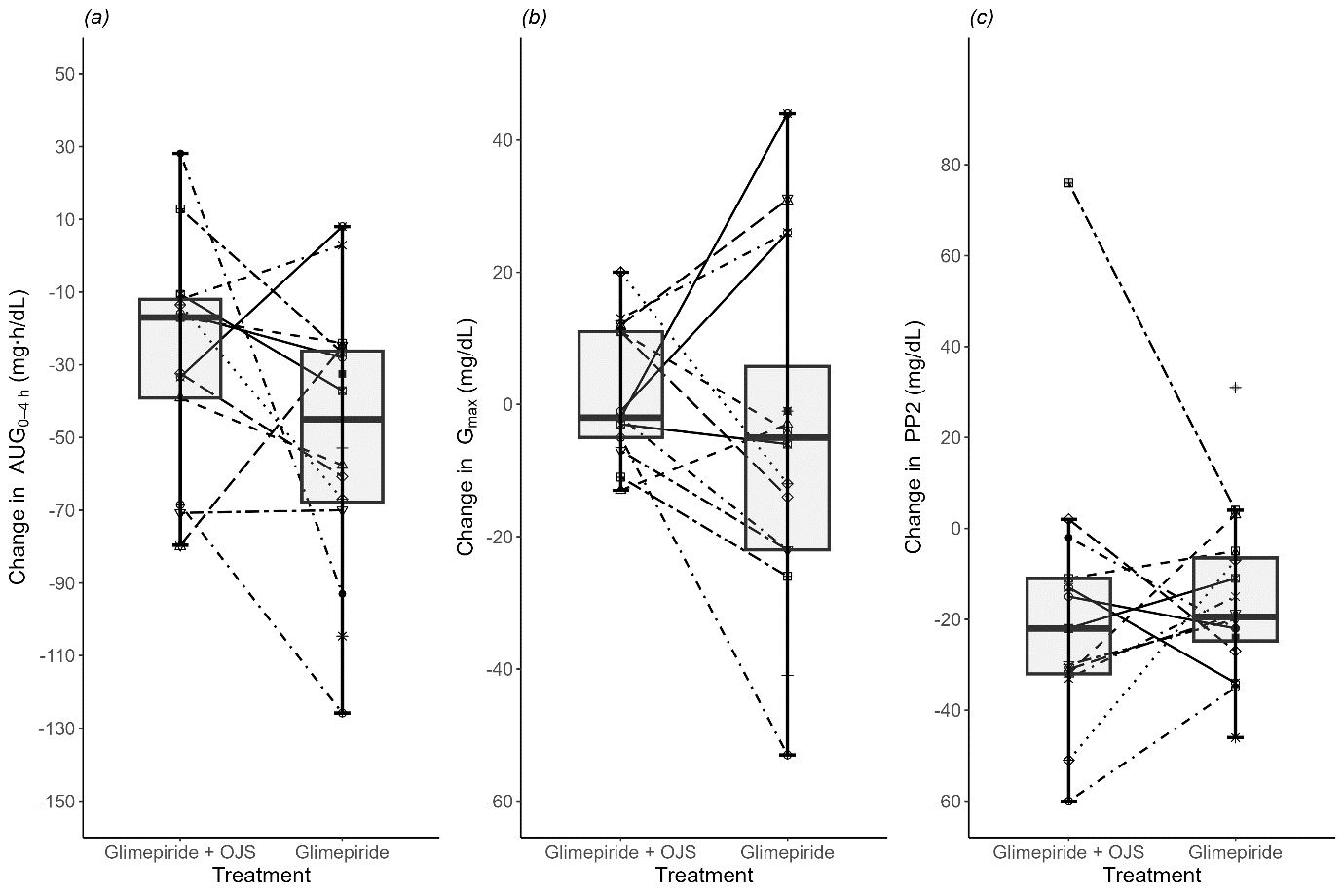


**Supplementary Fig. S6.** Individual change in (a) AUG_0–4 h_, (b) G_max_, and (c) PP2 for glimepiride alone or in combination with OJS. Box plots represent the lower quartile, median, and upper quartile. OJS, Ojeok-san; AUG_0–4 h_, area under the glucose concentration-time curve for 4 h after glucose administration; G_max_, maximum serum glucose concentration; PP2, 2-hour serum glucose.


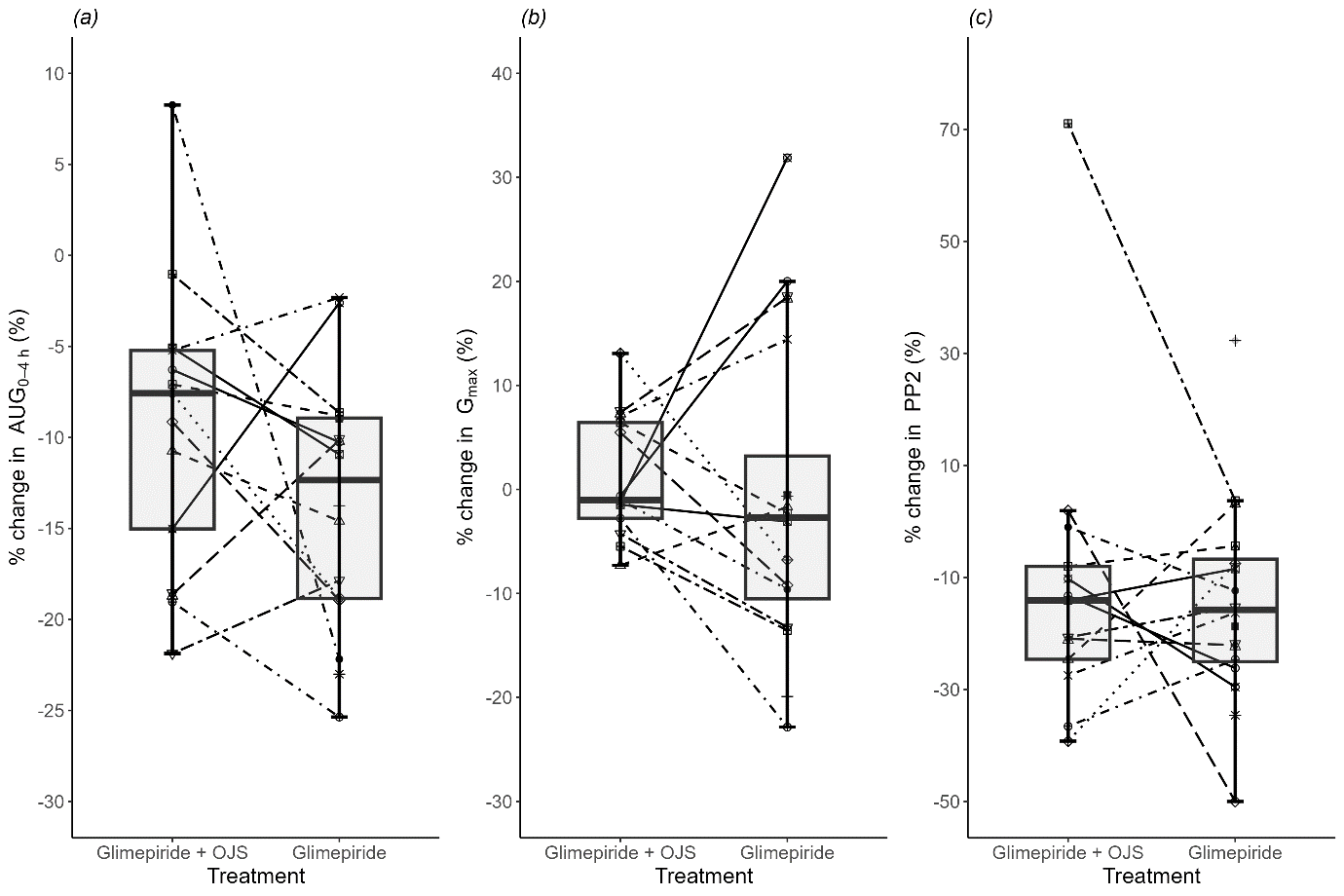


**Supplementary Fig. S7.** Individual percent change in (a) AUG_0–4 h_, (b) G_max_, and (c) PP2 for glimepiride alone or in combination with OJS. Box plots represent the lower quartile, median, and upper quartile. OJS, Ojeok-san; AUG_0–4 h_, area under the glucose concentration-time curve for 4 h after glucose administration; G_max_, maximum serum glucose concentration; PP2, 2-hour serum glucose.
